# The Effector Domain of MARCKS Is a Nuclear Localization Signal that Regulates Cellular PIP2 Levels and Nuclear PIP2 Localization

**DOI:** 10.1371/journal.pone.0140870

**Published:** 2015-10-15

**Authors:** Timothy D. Rohrbach, Nishi Shah, William P. Jackson, Erin V. Feeney, Samantha Scanlon, Robert Gish, Ryan Khodadadi, Stephen O. Hyde, Patricia H. Hicks, Joshua C. Anderson, John S. Jarboe, Christopher D. Willey

**Affiliations:** The Department of Radiation Oncology, The University of Alabama at Birmingham, Birmingham, AL, United States of America; University of Alabama at Birmingham, UNITED STATES

## Abstract

Translocation to the nucleus of diacylglycerol kinase (DGK)– ζ is dependent on a sequence homologous to the effector domain of Myristoylated Alanine Rich C-Kinase Substrate (MARCKS). These data would suggest that MARCKS could also localize to the nucleus. A single report demonstrated immunofluorescence staining of MARCKS in the nucleus; however, further experimental evidence confirming the specific domain responsible for this localization has not been reported. Here, we report that MARCKS is present in the nucleus in GBM cell lines. We then over-expressed wild-type MARCKS (WT) and MARCKS with the effector domain deleted (ΔED), both tagged with V5-epitope in a GBM cell line with low endogenous MARCKS expression (U87). We found that MARCKS-WT localized to the nucleus, while the MARCKS construct without the effector domain remained in the cytoplasm. We also found that over-expression of MARCKS-WT resulted in a significant increase in total cellular phosphatidyl-inositol (4,5) bisphosphate (PIP_2_) levels, consistent with prior evidence that MARCKS can regulate PIP_2_ levels. We also found increased staining for PIP_2_ in the nucleus with MARCKS-WT over-expression compared to MARCKS ΔED by immunofluorescence. Interestingly, we observed MARCKS and PIP_2_ co-localization in the nucleus. Lastly, we found changes in gene expression when MARCKS was not present in the nucleus (MARCKS ΔED). These data indicate that the MARCKS effector domain can function as a nuclear localization signal and that this sequence is critical for the ability of MARCKS to regulate PIP_2_ levels, nuclear localization, and gene expression. These data suggests a novel role for MARCKS in regulating nuclear functions such as gene expression.

## Introduction

MARCKS is an intrinsically unstructured protein that has been observed to influence numerous cellular processes including migration, proliferation, and survival [1–4]. It is well established that MARCKS circulates from the plasma membrane to the cytoplasm then back to the plasma membrane by reversible cycles of phosphorylation and de-phosphorylation; or by reversible cycles of calmodulin binding [5]. It is through this process by which MARCKS has been shown to reversibly sequester the phospholipid phosphatidyl-inositol (4,5) bisphosphate (PIP_2_). This process has been implicated in the regulation of the actin cytoskeletal dynamics, among other processes including spermatozoa acrosomal exocytosis [6], Akt signaling [1,7], and mitosis regulation [8]. These functions of MARCKS are mediated by a specific domain called the effector domain (ED). The ED contains four serines that are phosphorylatable by Protein Kinase C (PKC), 12 lysine residues, which sequester PIP_2_ by electrostatic interactions, and 5 phenylalanines that insert into the plasma membrane bilayer. Membrane association is also promoted by the presence of an N-terminal myristoylation sequence. The literature to date has focused on the sub-cellular localizations of MARCKS at the membrane and in the cytoplasm.

The MARCKS ED is homologous to a nuclear localization signal (NLS) in DGK-ζ that regulates translocation of DGK-ζ to the nucleus [9]. Phosphorylation of this domain in DGK-ζ prevents nuclear localization. A prior report showed immunofluorescence of various MARCKS mutant constructs expressed in 293 HEK cells demonstrating that MARCKS may also be present in the nucleus [10]. We have confirmed that MARCKS is indeed present in the nucleus in *Glioblastoma multiforme* (GBM) cells. Using an ED deleted mutant we have demonstrated that the critical domain for nuclear translocation is the MARCKS ED. We have found that this domain is crucial for regulating total cellular PIP_2_ levels, nuclear localization of PIP_2_, and gene expression. The data present here provides novel findings regarding MARCKS’ ability to migrate into the nucleus and regulate nuclear PIP_2_.

## Materials and Methods

### Cell culture

U87, U251 and D54 cells were obtained from Drs. Sontheimer and Benveniste and cultured as previously described [1]. U87, U251, and D54 glioma cells along with 293FT human embryonic kidney cells (Invitrogen) were cultured in Dulbecco’s modified Eagle’s medium (DMEM) with 10% fetal bovine serum (FBS), and 1% penicillin/streptomycin (Pen-Strep). All cells were maintained at 37°C in 5% CO_2_.

### MARCKS plasmid production

The ViraPower HiPerform T-REx Gateway Expression System (Cat. #A11141) and the pENTR221 entry vector (pENTR221-MARCKS) containing the wild-type (WT) MARCKS sequence were purchased from Invitrogen. The WT MARCKS sequence from pENTR221-MARCKS was cloned into the pLenti6.3/TO/V5-DEST destination vector using Clonase from the Gateway Expression System per manufacturer’s protocol to yield pLenti6.3/TO/V5-MARCKS-WT. Mutant MARCKS constructs were synthesized by and cloned into the pUC57 vector by GenScript (Piscataway, NJ). Deletion of the effector domain generated an effector domain deleted mutant (ΔED). Fragments from these plasmids containing the mutations were cloned into the pLenti6.3/TO/V5-MARCKS-WT via appropriate complementary restriction sites using standard protocols to generate the appropriate lentiviral expression vectors.

### Viral vector production

15x10^6^ 293FT cells were plated in 7ml of DMEM supplemented with 10% FBS without antibiotics onto a poly-D-lysine (PDL) (catalog # P7886, Sigma) coated 10cm dish. 36μl of Lipofectamine 2000 (catalog #11668, Invitrogen) was combined with 1.5 ml Opti-MEM media (catalog #11058, Invitrogen) and incubated for 5 minutes at room temperature. An additional aliquot of 1.5ml Opti-MEM media had 4μg of lentiviral packaging plasmid psPAX2 (Addgene plasmid 12260), lentiviral envelope plasmid PCMV-VSV-G (Addgene plasmid 8454), and appropriate lentiviral vector plasmid added. The plasmid and Lipofectamine media were mixed together and incubated for 20 minutes at room temperature. The Lipofectamine-plasmid mixture was added in a drop-wise fashion to the 293FT cells and incubated overnight. The following morning the media was replaced with fresh DMEM supplemented with 10% FBS and 1% Pen-Strep. Lentiviral supernatant was collected at 24 hours, filtered through a 0.45μm filter, aliquoted and stored at -80°C. QuickTiter p24 ELISA (Cell Biolabs, Inc.) was used to quantify lentivirus aliquots [1,4].

### Stable Cell Line Selection

5x10^5^ U87 cells were plated in 6 well plates and allowed to adhere overnight. The following morning, similar amounts of p24 quantified lentiviral particles containing tetracycline-repressor (Tet-R) plasmid along with 8 μg/ml polybrene were used to infect the cells. 500μl of virus were incubated with the cells for 2 hours at 37°C in 5% CO_2_. After 2 hours incubation, media was removed and fresh media was added for 3 days at which point 500μg/ml geneticin (G418, Life Technologies) was added to the media. After 3 passages in geneticin, U87 Tet-R expressing cells were frozen for stock and were re-plated on 6 well plates at 5x10^5^ cells/well to be infected with virus particles containing MARCKS mutants. Identical procedure as described above was performed for virus infection, however after 3 days of culture, cells were selected for with 1μg/ml blastacidin (Life Technologies). After 3 successful passages, cells were frozen down for stock or used in experiments. To over-express WT or ΔED MARCKS expression 1μg/ml doxycycline was added to the culture media overnight.

### Western Blot

Briefly, cells were lysed using MPER lysis buffer (Thermo #78501) supplemented with 5% protease (Sigma #P8340) and 1% phosphatase inhibitors (Phosphatase Inhibitor 2 -Sigma P5726 and Phosphatase Inhibitor 3—Sigma P0044). Between 25–50 μg of protein for each sample were separated by electrophoresis through an 8% SDS-polyacrylamide gel and transferred to a PVDF membrane (Immobilon). Blots were blocked in 5% milk, 1% bovine serum albumin (BSA), tris-buffered saline and tween 20 (TBST) for 1 hour at room temperature. Blots were incubated overnight at 4°C and probed with the following primary antibodies: MARCKS (1:1000, Abcam, Ab52616), actin (1:1000, Santa Cruz, sc-1616), V-5 (1:5000, Invitrogen, R96125), lamin A/C (1:1000, Santa Cruz, sc-6215), α-Tubulin (1:2000, Santa Cruz, sc-53646). After overnight incubation, blots were washed 3 x 5 min washes in TBST. Approximately 1:5000 dilution of the appropriate secondary antibodies in 5% milk, 1% BSA, TBST were used and blots were incubated for 1 hour at room temperature. Bands were detected via a chemiluminescence approach as described previously and following PerkinElmer’s instructions (Western Lightning *Plus* ECL, NEL102001EA) [1].

### Immunofluorescence

Glass cover slips were coated with 50μg/ml PDL for 2 hours at 37°C. 10,000 cells were plated on each cover slip and attached overnight. For MARCKS over-expression studies, 1μg/ml doxycycline was added to the culture for 21 hours. Cells were fixed for 15 minutes with 4% paraformaldehyde, and then permeabilized with 0.25% triton-X100 + 1% BSA + 0.3M glycine PBS for 30 minutes. Cells were blocked for 30 minutes in 1% BSA + 0.3M glycine. Anti-MARCKS antibody (1:500, Abcam, ab55451) was used with AlexaFluor488 (1:1000, Invitrogen, A11029). V-5 (1:200, Invitrogen, R96125) primary was added followed by anti-mouse AlexaFluor594 (1:500, Invitrogen, A11032). PIP_2_ (1:400, Santa Cruz, sc-53412) was conjugated with Thermo DyLight Microscale Antibody Labeling Kit, (DyLight 488, #53025) according to manufacturer protocols. Lastly, 300ng/ml DAPI (4’,6-Diamidino-2-Phenylindole) was used to stain cellular DNA. Coverslips were then mounted on slides using Aqua PolyMount (PolyScience, #18606). Images were captured on Zeiss LSM 710 Confocal Laser Scanning Microscope or Nikon A1 High Speed Advanced Confocal Microscope in the UAB High Resolution Image Facility.

### Total PIP_2_ ELISA

Whole cell lysates of U87 WT and ΔED mutants were collected and PIP_2_ levels were quantified using PI(4,5)P_2_ Mass ELISA Kit according to manufacturer’s specifications (Echelon Biosciences Inc, Salt Lake City, Utah) [11]. GraphPad Prism (GraphPad Software) was used for statistical analysis and to graph data.

### Nuclear PIP_2_ Quantification by Immunofluorescence

The protocol for quantification of nuclear PIP_2_ staining was adapted from a previously published protocol by ImageJ [12]. Cells were treated and stained as discussed above in the Immunofluorescence section. The cells were imaged as above by three channels together as Red (MARCKS)–Green (PIP_2_)–Blue (DAPI). The image was then split into three separate channels using the RGB merge/split function. Each nucleus was traced by the DAPI stain from the blue channel using the trace tool and defined as a region of interest (ROI). Using the measure option in the program’s ROI Manager the integrated density value (IDV) of the blue channel was obtained. Then, to quantify the stain for PIP_2_ per nucleus the image calculator from the process menu was used to create a merged image combining the green (PIP_2_) channel with the blue channel (DAPI) using the operator “AND.” The IDV of the blue and green channel was then obtained as well. Then, the IDV of the blue channel was subtracted from that of the blue and green channel to obtain the IDV of the green channel, which represents the intensity of PIP_2_ stain for that particular nuclei. 19 nuclei were quantified from the ΔED construct and 18 nuclei were quantified from the WT-MARCKS construct. The results are presented as the mean IDV of the green channel per nucleus.

### RNA Expression Analysis

The nCounter GX Human Cancer Reference Kit (NanoString Technologies; Seattle, WA) was used for RNA expression. U87 WT-MARCKS and ΔED-MARCKS cells were induced with doxycycline overnight with RNA collected the following day. A total of 100ng of RNA was prepared in 30 μl reaction volume and run on the automated nCounter system. Raw data was quality controlled and normalized using positive and negative control spots on the chip as well as 5 housekeeping reference genes [13,14].

### Proteomic Analysis

The Kinex KAM-850 antibody microarray (Kinexus Bioinformatics Corporation, Vancouver, British Columbia, Canada) was utilized for investigating differences in protein levels as well as levels of phosphorylated proteins. U87 WT-MARCKS and ΔED-MARCKS cells were induced with doxycycline overnight. Cellular lysate was collected and prepared according to manufacturer’s protocols. Kinexus Bioinformatics Corporation performed the analysis of the microarray [15].

## Results

### MARCKS localizes to the nucleus in GBM cells

**Fig 1:**
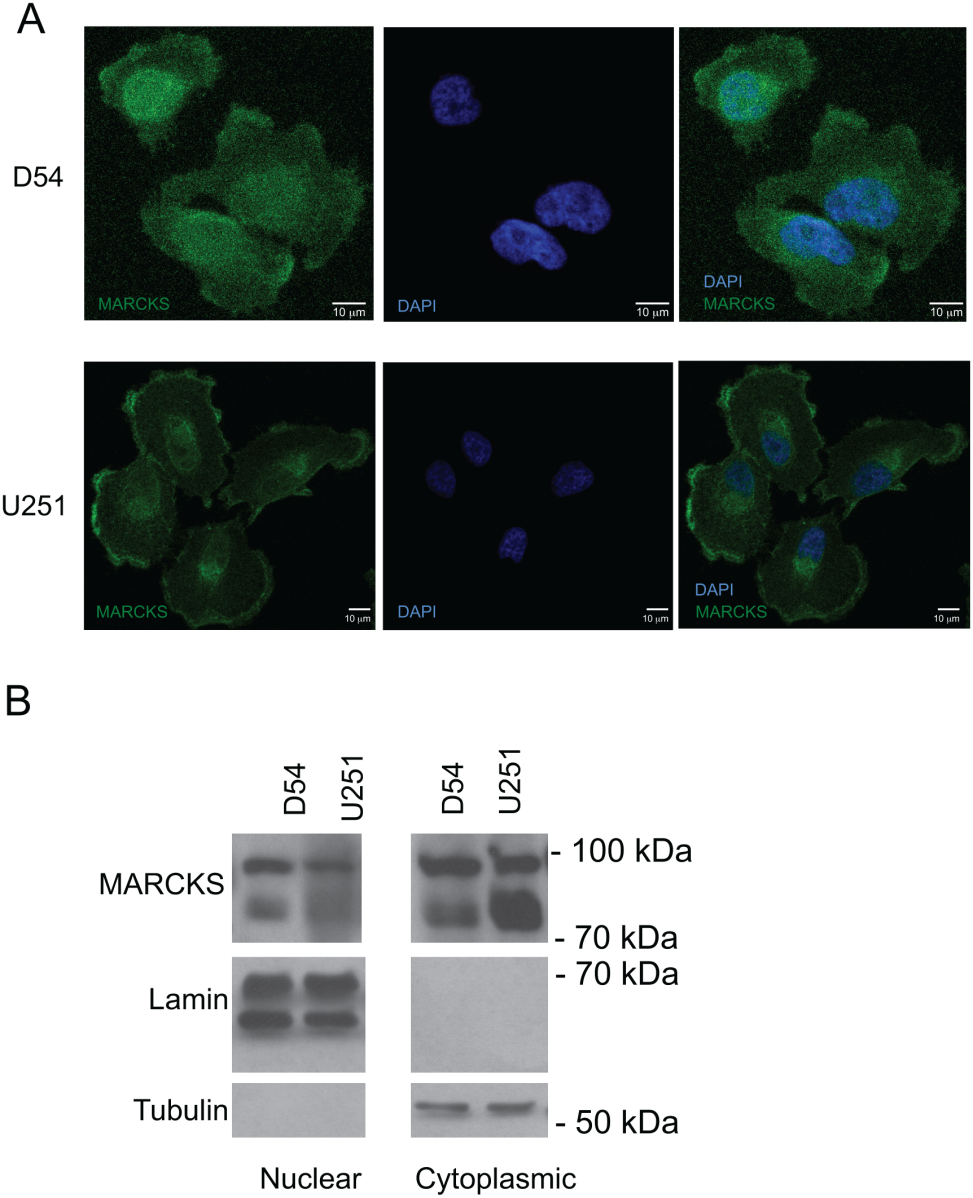
MARCKS localizes to the nucleus in D54 and U251 glioma cells. **A)** D54 and U251 cells were fixed and stained for MARCKS and DAPI with the merged image shown on the right side as indicated. **B)** D54 and U251 cell lysates were separated into nuclear and cytoplasmic fractions. The proteins were then were separated by 8% SDS-PAGE and probed for MARCKS, the cytoplasmic marker tubulin, and the nuclear marker, lamin.

We have been investigating MARCKS within GBM model systems based on our previous work that identified MARCKS as a regulator of GBM growth and radiation sensitivity [1]. As part of our subsequent studies, we were particularly interested in the subcellular localization of MARCKS as its role in cell biology is felt to be heavily influenced by whether MARCKS is plasma membrane bound or within the cytoplasm. However, when we examined MARCKS localization in GBM cells by immunofluorescence, we made the observation that a substantial portion of MARCKS localized to the nucleus (Fig 1A). Indeed, in D54 cells and to a lesser extent, in U251 cells, we identified MARCKS colocalizing to the DAPI-stained nucleus, which we confirmed by Western blotting of nuclear and cytoplasmic fractions using lamin and tubulin as nuclear and cytoplasmic subcellular markers, respectively (Fig 1B) [16].

### The MARCKS Effector Domain has homology with known nuclear localization sequences

**Fig 2:**
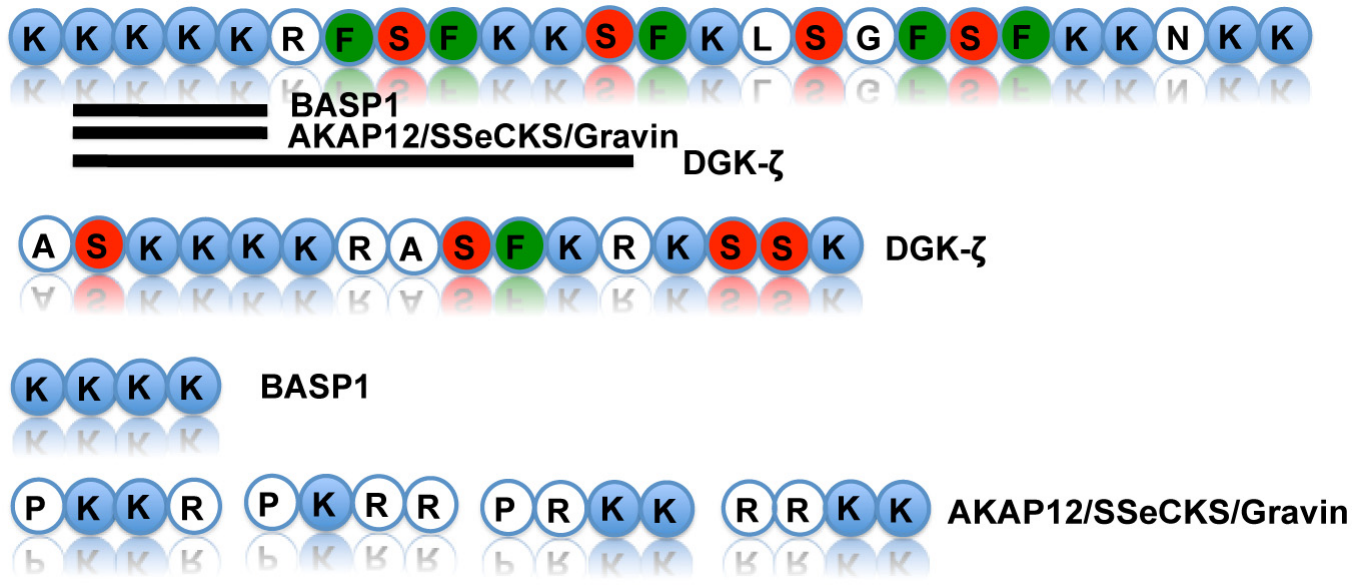
MARCKS Effector Domain contains high homology with other putative nuclear localization sequences. A schematic of the amino acid sequence of the MARCKS ED with alignment to the nuclear localization sequences of DGK-ζ, BASP1, and AKAP12/SSeCKS/Gravin. Blue circles represent lysines, green circles represent phenylalanines, red circles represent serines, while white circles represent other amino acids using single letter abbreviations. The black lines under the MARCKS effector domain amino acid sequence represent sequence homology to DGK-ζ, BASP1, and AKAP12/SSeCKS/Gravin, respectively.

Based on the novel findings of MARCKS in the nucleus of cancer cells, we examined the literature and MARCKS amino acid sequence to try to identify a potential nuclear localization sequence (NLS). The sequence of the 25 amino acids that are contained in the ED region of MARCKS are: KKKKKRFSFKKSFKLSGFSFKKNKK (Fig 2) [17]. DGK-ζ has a 16 amino acid sequence (ASKKKKRASFKRKSSK), which resembles MARCKS’s ED [9]. In addition to DGK-ζ, Brain acid-soluble protein 1 (BASP1) and A kinase anchoring protein 12 (AKAP12/SSeCKS/Gravin) have putative nuclear localization sequences that are homologous to the ED region of MARCKS. BASP1 is a protein that contains an N-terminal myristoylation domain along with conserved serine residues that can be phosphorylated by PKC. BASP1 is able to bind to PIP_2_ and translocates to the nucleus with a putative nuclear localization sequence KKKK [18,19]. AKAP12 is also a protein with a myristoylation addition along with 5 putative NLS. Streb et al. described four of the NLS: PKKR, PKRR, PRKK, RRKK [20]. The NLS of DGK-ζ is homologous to the sequence of the MARCKS ED. The lysine rich NLS of BASP1 matches identically with MARCKS ED. The AKAP12 NLS has lysine residues with arginine residues that would closely resemble the KKKK sequence in terms of structure and charge. This information supports that the ED region of MARCKS contains a putative NLS.

### The Effector Domain is required for nuclear localization of MARCKS in U87 cells

**Fig 3:**
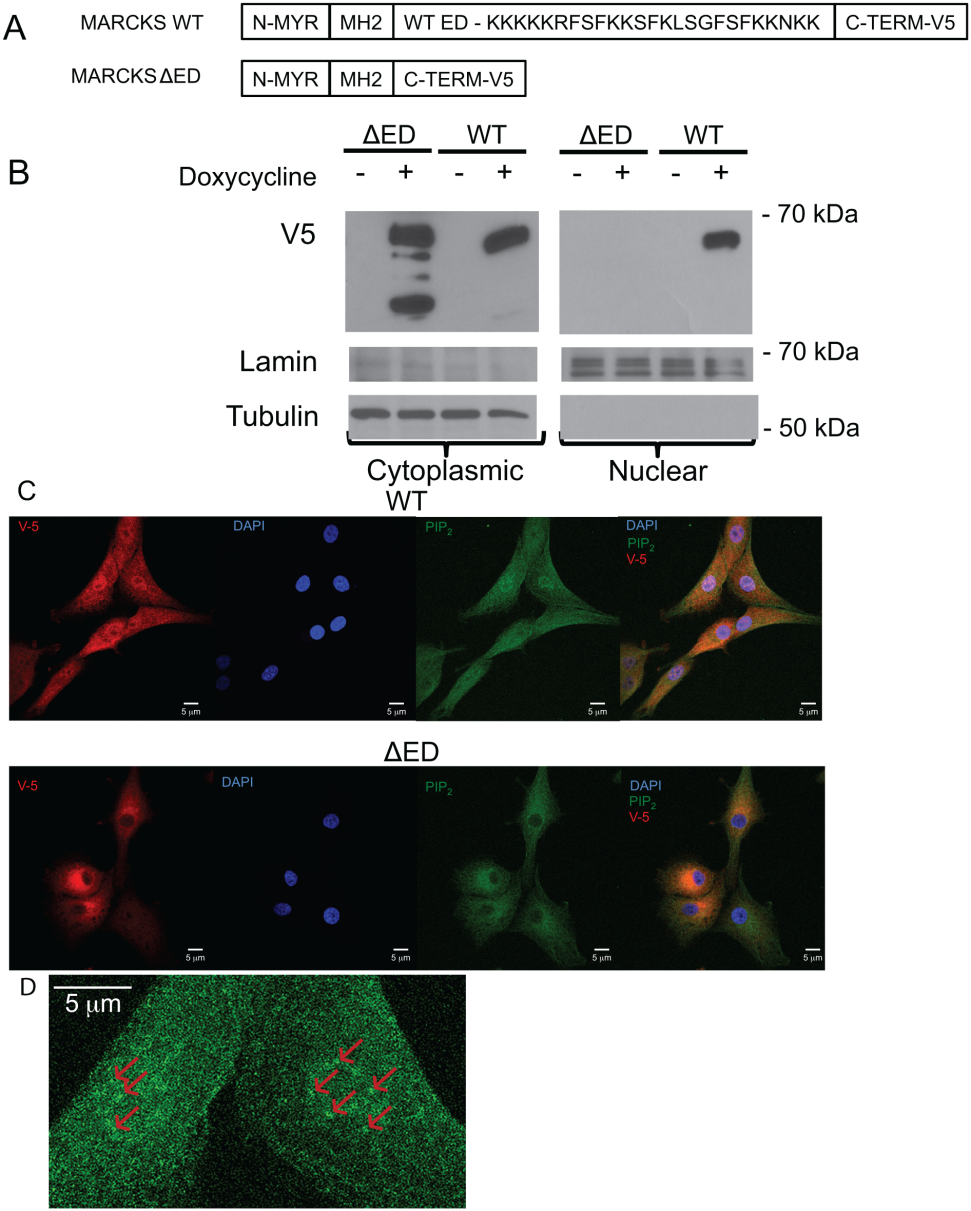
MARCKS mutant expression and localization in U87 glioma cells. **A)** Diagram depicting the domains of the engineered MARCKS wild-type (WT) and effector domain deleted (ΔED) lentiviral constructs. Starting at the N-terminus there is a myristoylation domain (N-MYR), MH2 domain (N-MH2), an effector domain (WT-ED) with amino acid sequence indicated, and lastly a C-terminus V-5 tag. **B)** Western blot showing doxycycline inducible MARCKS mutant over-expression in U87 cells. Nuclear and cytoplasmic fractions were prepared and separated by SDS-PAGE and probed for V-5 (for MARCKS expression), lamin and tubulin for nuclear and cytoplasmic fraction, respectively. **C)** Confocal microscopy is shown for U87 WT-MARCKS (WT) and ΔED-MARCKS (ΔED) cells were fixed and stained for MARCKS (V5), nucleus (DAPI), and PIP_2_ with the merged image shown on the right. **D)** High magnification of the PIP2 staining of the nucleus with punctate staining marked with red arrows.

U87 GBM cells were selected for our subsequent studies due to their low endogenous MARCKS expression [1]. To determine whether the ED was required for nuclear localization, we generated a WT-MARCKS, an ED deleted-MARCKS (ΔED), as well as an empty control lentivirus and infected Tet-R expressing U87 GBM cells to produce a doxycycline inducible expression system. The two MARCKS constructs contained V5 C-terminal tags to distinguish exogenous from endogenous expression (Shown schematically in Fig 3A). We then induced the WT, ΔED, and control virus infected U87 cells with doxycycline and then performed subcellular fractionation to isolate a cytoplasmic and nuclear fraction as in Fig 1B. As shown in Fig 3B, WT-MARCKS, but not the ΔED-MARCKS, was detected in the nuclear fraction as evidenced by V5 Western blot. Interestingly, a prominent smaller molecular weight band is noted in the ΔED-MARCKS expressing cells. This is consistent with prior studies showing that MARCKS is a target of proteases such as Cathepsin B [21]. Since the V5 tag is on the C-terminus, we anticipate this fragment is produced by an N-terminal clipping event. As before, tubulin and lamin Western blots confirmed the subfraction identities.

To confirm that the MARCKS ED was required for MARCKS nuclear localization, we performed anti-V5 immunofluorescence of doxycycline-induced MARCKS mutant overexpression in U87 cells (Fig 3C). Whereas WT-MARCKS demonstrated cytoplasmic and nuclear staining, the ΔED-MARCKS was excluded from the nucleus.

We confirmed further our findings in U251 cells (S1 Fig) where we observed that the ΔED-MARCKS did not localize to the nucleus. Moreover, when the ED serine amino acids were changed to aspartic acid, mimicking phosphorylation, or alanine amino acids, mimicking a non-phosphorylatable state, MARCKS was still able to migrate into the nucleus. These data confirm that the ED of MARCKS is required for its nuclear translocation. It should be noted that there are some molecular weight differences between the endogenous MARCKS in Fig 1 and the V5-tagged MARCKS in Fig 3. MARCKS Western blot migration based on amino acid sequence is predicted to migrate around 30 KDa. However, because MARCKS is an intrinsically unstructured protein, cell line (and antibody) differences lead to MARCKS typically migrating between about 60–90 KDa. To confirm that the results seen were not due to problems with high levels of overexpression, we isolated additional single cell clones for WT-MARCKS and ΔED-MARCKS mutants in which WT-MARCKS was more highly expressed and confirmed that WT-MARCKS could also produce multiple bands which did not prevent nuclear localization (S2 Fig).

### Total PIP_2_ levels increase in WT MARCKS U87 cells and localize to the nucleus

**Fig 4:**
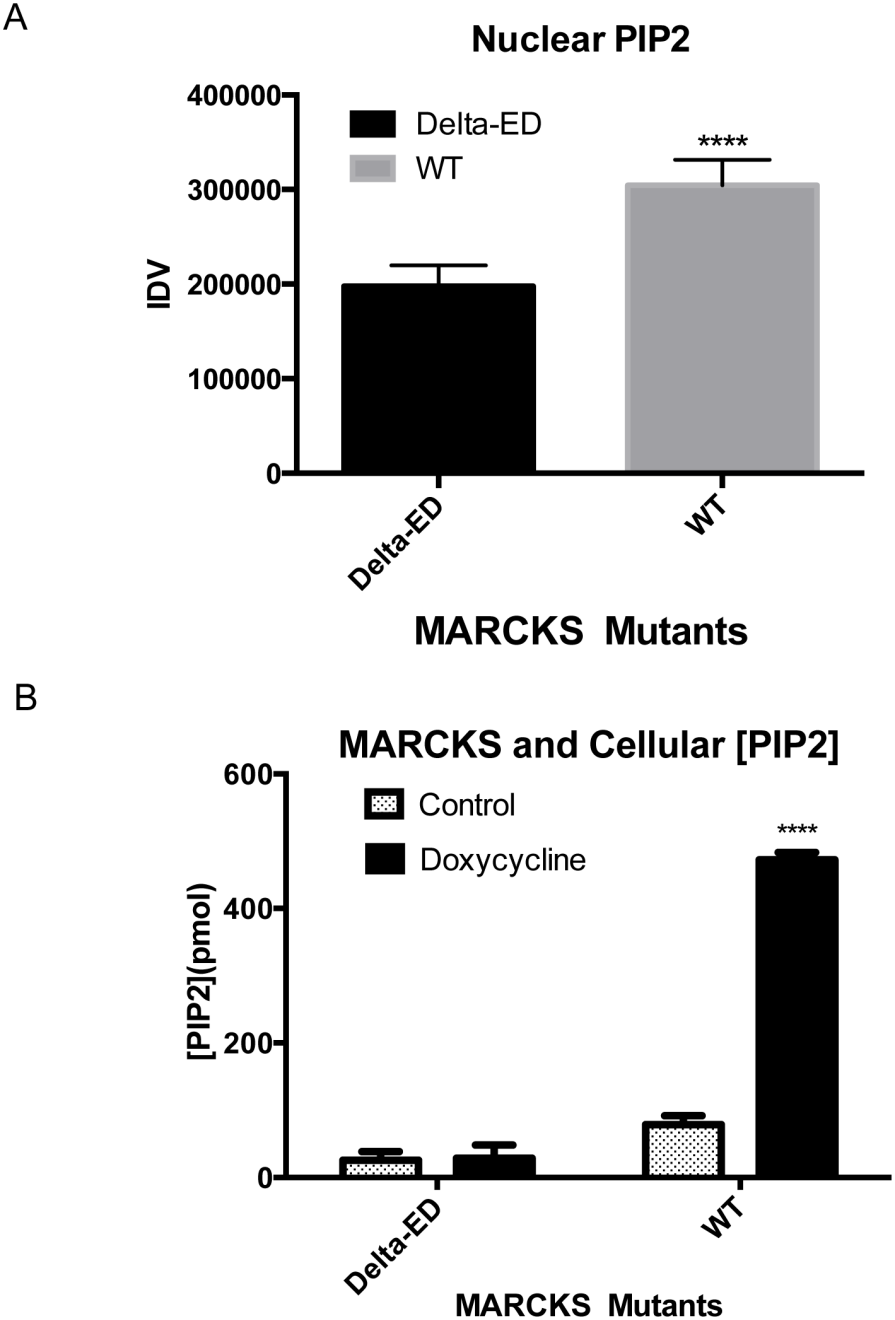
Changes in PIP_2_ in ED-deleted MARCKS expressing glioma cells. **A)** Nuclear PIP_2_ immunofluorescence staining was quantified in the U87 WT- and ΔED- MARCKS expressing cells as described in Materials and Methods. Briefly, DAPI-defined nuclear regions of interest were generated and an integrated density value (IDV) was calculated for each nucleus. The mean IDV and standard error are shown (t-test p = 0.0043). B) Total levels of PIP_2_ were quantified for U87 WT- and ΔED- MARCKS expressing cells with or without doxycycline treatment using a PI(4,5)P_2_ Mass ELISA KIT. Mean PIP_2_ concentrations with standard errors are shown (t-test p< 0.0001).

Because MARCKS has the capacity to bind and sequester PIP_2_ through its ED, we performed immunofluorescence staining of PIP_2_ in U87 WT-MARCKS and ΔED-MARCKS cells as shown in Fig 3C. We noted that doxycycline-induced overexpression of WT-MARCKS caused an increase in PIP_2_ staining, particularly in the nucleus (punctate pattern shown in Fig 3D, similar to previous reports [19]). We quantified the PIP_2_ staining and found a significant difference (p = 0.0043) in nuclear PIP_2_ staining between WT-MARCKS and ΔED-MARCKS expressing U87 cells (Fig 4A). To confirm that the ED is required for modulating PIP_2_ levels, we performed a PIP_2_ mass ELISA on total cellular PIP_2_ concentration (Fig 4B). Doxycycline-induced ΔED-MARCKS did not significantly alter PIP_2_ levels (Fig 4B) while WT-MARCKS overexpression promoted a significant increase in total cellular PIP_2_ levels (p< 0.0001).

### Changes in Cell Signaling

**Fig 5:**
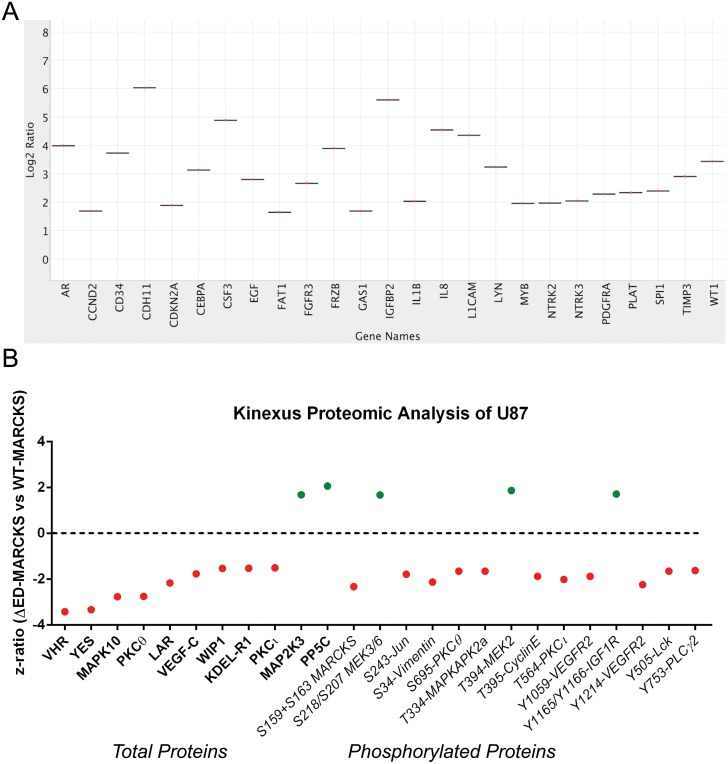
Changes in RNA expression and protein levels with ED-deleted MARCKS. **A)** After overnight doxycycline-induction of ΔED-MARCKS or WT-MARCKS U87 cells, RNA was collected and ran on NanoString nCounter GX Human Cancer Reference Kit. Data was analyzed on nSolver software. Violin plot displays > 1.5 log fold increase in GBM cells overexpressing an ΔED-MARCKS protein as compared to WT MARCKS. **B)** Changes in total protein levels as well as phosphorylated protein levels between the ΔED and WT- MARCKS expressing U87 GBM cell lines were determined by Kinex KAM-850 microarray. Data is displayed as z-ratio of ΔED results compared to WT-MARCKS results. Increase in z-ratio is indicated in green dots while decrease is indicated in red dots. Total protein targets are listed in standard font while phosphorylated proteins are listed in italicized font with phosphorylated amino acids designated.

With MARCKS being able to migrate into the nucleus and bind PIP_2_ it is possible that MARCKS is able to influence gene expression [22,23]. The nCounter GX Human Cancer Reference Kit (NanoString Technologies) was used to detect expression differences in 230 cancer-related genes (S1 Table). WT-MARCKS and ΔED-MARCKS were doxycycline-induced overnight and RNA was collected the following day. Fig 5A displays a violin plot with 32 genes having a 1.5 log overexpression when MARCKS was not able to migrate into the nucleus.

In addition to changes in gene expression, differences in protein levels as well as phosphorylated proteins were observed between ΔED-MARCKS and WT-MARCKS U87 cells (Fig 5B and S2 Table). Over-expression of ΔED-MARCKS lead to a reduced level of several phosphoproteins and kinases. However, some proteins were positively regulated by ΔED-MARCKS (shown in green).

## Discussion

Our study shows for the first time, endogenous MARCKS being present in the nucleus, specifically in GBM cells. We sought to determine the location of the NLS and the impact that nuclear MARCKS might have on nuclear PIP_2_ regulation. A prior study by Topham et al. suggested that a sequence homologous to the ED of MARCKS in DGK-ζ can function as a NLS. The authors demonstrated that removal of the putative NLS (ASKKKKRASFKRKSSK) in DGK-ζ, attenuated nuclear localization of the protein [9]. Further support for this region functioning as an NLS comes from studies where mutation of the basic amino acid rich sequence of proteins BASP-1 and AKAP12/SSeCKS/Gravin prevented them from entering the nucleus [19,20]. When we deleted the ED region of MARCKS (KKKKKRFSFKKSFKLSGFSFKKNKK), which contains high homology to the NLS of DGK-ζ, BASP-1, and AKAP12, MARCKS did not localize to the nucleus as indicated by our immunofluorescence and sub-cellular fractionation experiments. This appears to be independent of the phosphorylation status of MARCKS as mutations of the serine residues of the ED to alanine or aspartic acid did not prevent nuclear localization (S1 Fig), which is in contrast to what was shown for DGK-ζ [9]. Moreover, we believe that this is independent of overexpression levels. While Fig 3 clearly shows that the MARCKS WT clone has a lower amount of expression as compared to the MARCKS ΔED, we isolated other single cell clones for both MARCKS-WT and MARCKS-ΔED in which the WT was more highly expressed. While the higher MARCKS-WT expression did produce a similar banding pattern as seen for MARCKS-ΔED from Fig 3, this did not impact MARCKS-WT’s ability to migrate to the nucleus (S2 Fig). These data confirm that the ED of MARCKS does indeed function as a NLS.

MARCKS was first reported in the literature to be in the nucleus in a study on MARCKS function in cell spreading in 293 cells [10]. The authors utilized various MARCKS ED serine mutant constructs (namely to alanine, asparagine and aspartic acid) as well as mutation of the myristoylation site in the N-terminus (N-Myr). The authors found that when highly overexpressed, the two N-Myr deficient mutants could be seen in the nucleus. They hypothesized that the loss of the myristoylation moiety allowed translocation to the nucleus. Other authors have found a similar result where loss of myristoylation led to increased nuclear localization of BASP1, however; they also found that myristoylation was critical for the nuclear function of BASP1 [19]. Therefore the myristoylation moiety in itself does not exclude BASP1 from the nucleus. We utilized a wild-type MARCKS construct with the myristoylation signal intact and it localized to the nucleus, while the construct with the ED deleted did not. Interestingly, one of the mutants that Spizz and Blackshear noted to be in the nucleus was a double mutant myristoylation deficient and pseudo-phosphorylated MARCKS in which the serines were mutated to aspartic acids. This result is somewhat unexpected as it is known that phosphorylation of the MARCKS ED in DGK excludes it from the nucleus [9]. Aspartic acid has a single negative charge while a phosphate has three, so it is quite possible that the decrease in negative charge and associated decrease in steric hindrance for protein interactions is not enough to disrupt the NLS. However, we found that serine mutations to aspartic acid or alanine did not prevent nuclear localization. This is consistent with a report from mouse oocytes in which phosphorylated MARCKS could localize to the nucleus [24]. Taken together, these studies suggest that the ED is in fact the critical domain that regulates nuclear localization of MARCKS and that the N-Myr may play a role in regulating the amount of localization. However, to completely characterize the putative NLS sequence in MARCKS would require additional point mutations to determine which of the 12 lysine residues and 5 phenylalanines are absolutely required for nuclear localization, which is beyond the scope of this paper.

Importantly, we also found that MARCKS can regulate total as well as nuclear PIP_2_ levels and localization. MARCKS binds to and sequesters PIP_2_ by electrostatic interactions of the basic lysine residues in the ED with the acid phosphate groups of PIP_2_. In this sequestered state, PIP_2_ is not available for modifications by other proteins such as PLC or PI3K. Therefore, we hypothesized that increased expression of MARCKS would lead to higher levels of cellular PIP_2_, while deletion of the ED would have no effect. This was confirmed by our PIP_2_ ELISA, which demonstrated increased levels of cellular PIP_2_ with expression of WT-MARCKS, but not with the ED deleted. This is consistent with the recent finding that low MARCKS levels in the hippocampus produces low PIP_2_ levels and reduced cognition in aging mice [25]. We also found that there was increased staining for PIP_2_ in the nucleus when WT-MARCKS was over-expressed compared to the ED deleted mutant, consistent with the ability of similar proteins such as BASP1 to regulate nuclear localization of PIP_2_ [19].

The importance of PIP_2_ sequestration by an N-myristoylated protein with a basic amino acid rich effector domain has previously been reported to be important for gene regulation by recruitment of PIP_2_. Prior studies with the protein BASP1 showed that N-myristoylation and PIP_2_ recruitment to nuclear speckle domains plays an important function in the transcriptional repression of the Wilm’s Tumor gene (WT1) [19]. This repression occurs through the recruitment of histone deacetylase enzymes (HDACs) with BASP1. Our finding that WT-MARCKS but not ΔED-MARCKS localizes to the nucleus with PIP_2_ suggests that MARCKS may potentially function in a similar fashion as BASP1 in the nucleus by altering gene expression. Our initial investigation revealed that 32 genes had increase expression when MARCKS was not present in the nucleus. These genes were involved in pro-cancer related functions including cell cycle progression, adhesion, proliferation, and survival. Further studies showed changes in protein as well as phosphorylated protein levels when MARCKS’s ED was deleted. Studies are ongoing to determine what the function of MARCKS is in the nucleus and how it might impact cancer cell biology.

## Supporting Information

S1 Fig MARCKS mutant subcellular fractionation in U251 cells. Western blots of cytosolic and nuclear subfractions of U251 cells expressing doxycycline induced wild type (WT), non-phosphorylatable (NP) in which serine residues of the effector domain (ED) were mutated to alanine, a pseudo-phosphorylated (PP) in which the serine residues of the ED were mutated to aspartic acids, and ED-deleted (ΔED) MARCKS containing a V5 tag. Western blots of V5 and Lamin are shown.
(EPS)

S2 Fig MARCKS mutant expression and localization in U87 glioma cells. Western blot showing another set of single clones of doxycycline-inducible MARCKS mutants in U87 cells. Nuclear and cytoplasmic fractions were prepared and separated by SDS-PAGE and probed for V-5 (for MARCKS expression).
(TIF)

S1 Table Nanostring Data. Log2 Ratios of normalized gene expression from NanoString nCounter GX Human Cancer Reference Kit (NanoString Technologies; Seattle, WA) for effector domain deleted (dED) and wild type (WT) MARCKS expressing cells.
(CSV)

S2 Table Kinex KAM-850 Data. The Kinex Kam-850 antibody microarray (Kinexus Bioinformatics Corporation, Vancouver, British Columbia, Canada) was used to compare differences in proteins and phosphoproteins between ΔED-MARCKS and WT MARCKS expressing cells.
(XLSX)
