# Supplementary figures and images for: The Effector Domain of MARCKS Is a Nuclear Localization Signal that Regulates Cellular PIP2 Levels and Nuclear PIP2 Localization
Source: PLoS One. 2015 Oct 15;10(10):e0140870. doi: 10.1371/journal.pone.0140870 (PMC4607481; doi:10.1371/journal.pone.0140870)

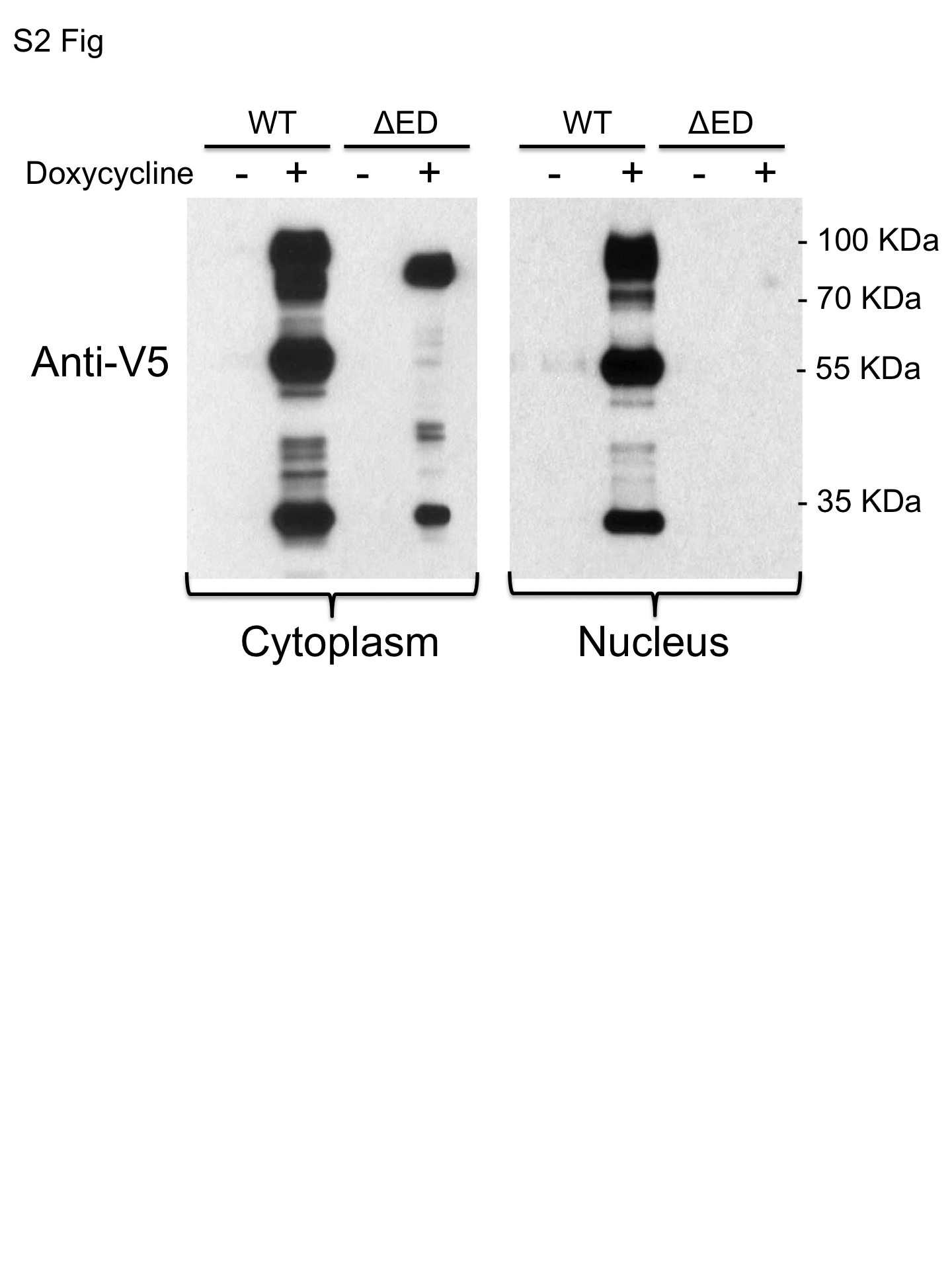

Supplement: S2 Fig — Western blot showing another set of single clones of doxycycline-inducible MARCKS mutants in U87 cells. Nuclear and cytoplasmic fractions were prepared and separated by SDS-PAGE and probed for V-5 (for MARCKS expression). (TIF) [file pone.0140870.s002.tif]
